# Cadmium Uptake and Relationship to Feeding Habits of Freshwater Fish from the Ayeyarwady River, Mandalay, Myanmar

**DOI:** 10.5696/2156-9614-10.26.200608

**Published:** 2020-05-26

**Authors:** Khin Myint Mar

**Affiliations:** Department of Zoology, University of Magway, Magway, Myanmar

**Keywords:** cadmium, fish muscle, Ayeyarwady River, Myanmar

## Abstract

**Background.:**

Pollution of the aquatic ecosystem by heavy metals is increasing due to anthropogenic activities. Cadmium (Cd) can accumulate in soil, be taken up by plants, and passed on in the food chain to animals and humans.

**Objectives.:**

The present study was conducted to analyze the uptake of Cd in muscles of sampled fish with different feeding habits and to compare levels of Cd in fish from the Ayeyarwady River, Myanmar with international standards.

**Methods.:**

The acid digestion procedure was used for sample preparation. Cadmium concentrations in fish samples were determined by flame atomic absorption spectrophotometry (Perkin Elmer AAanalyst 800 and Winlab-32 software).

**Results.:**

In herbivorous fish species, Cd content ranged from 0.07 (Catla catla) to 0.086 mg/kg (Osteobrama belangeri). In carnivorous fish species, Cd ranged from 0.060 (Mystus leucophasis) to 0.083 mg/kg (Wallago attu). In omnivorous fish species, Cd ranged from 0.07 (Botia histrionica) to 0.084 mg/kg (Gudusia variegata). Cadmium content did not differ significantly across the three types of feeding habits (p>0.05).

**Discussion.:**

The accumulation of Cd in the muscle of studied fish was lower than the permissible limit set down by the European Union in 2001 (0.1 ppm), but above the limits set down by the Food and Agriculture Organization/World Health Organization, European Commission (0.05 ppm) and within the limit of United States Food and Drug Administration (0.01–0.21 ppm). The data obtained in the present study indicate that levels of Cd were not significantly different across fish species with different feeding habits.

**Conclusions.:**

The examined fish samples were not fully safe for human consumption due to high levels of Cd.

**Competing Interests.:**

The authors declare no competing financial interests.

## Introduction

Pollution of the aquatic ecosystem by heavy metals is increasing due to anthropogenic activities. Aquatic pollution is growing at an alarming rate and threatens conservation ecology and public health.^1^ This situation has further intensified due to increases in population, urbanization, industrialization, and agricultural practices.^2^,^3^ Long-term simultaneous application of fertilizer and manure in agricultural areas has been linked with higher metal accumulation in soil and plants.^4^,^5^ In some fertilizers, the presence of cadmium (Cd) at high concentrations is of most concern due to the toxicity of this metal and its ability to concentrate in soils and bioaccumulate in plants and animals.^6^,^7^ Most Cd is released from human activities such as mining and smelting of sulfide ores, fuel combustion, and application of phosphate fertilizers or sewage sludge.^8^

Fish is consumed globally as a source of nutrients, particularly protein. Fish flesh contains high quality protein and high content of two types of omega-3 polyunsaturated fatty acids. Therefore, fish consumption is rising among an increasingly health-conscious population.^9^,^10^

Cadmium acts as a fish stressor, leading to metabolic alterations and decreasing total protein concentrations.^11^ One route of exposure of Cd to humans is through the consumption of fish contaminated with Cd accumulating in the human body, especially in the kidneys, leading to kidney dysfunction with impaired reabsorption of proteins, glucose and amino acids, etc.^12^

Myanmar is an agricultural country. According to informal interviews with local farmers and information available at agricultural centers, the application of phosphate fertilizers in this area is greater than land requirements, which may result in high concentrations of Cd in soil.^13^ In 2007, Robinson reported that the Ayeyarwady River had been declining in water quality for many years.^14^ Siltation occurs at a rate of 360 million tons annually, ranking the third highest in the world, due to mining operations, deforestation, lack of soil protection and land overexploitation. This poses a major threat to the river. Moreover, the agricultural sector also contributes to impacts on river water quality.^14^ Uncontrolled use of fertilizers and lack of runoff treatment mechanisms in agricultural areas may have contributed to this increase. Direct sewage disposal, landfills, organic wastes and manure are related to high ammonia nitrogen concentration in rivers.^15^ In light of these issues, the present study was conducted to analyze the uptake of Cd in muscles of fish species across different feeding habits and to assess the level of Cd in fish muscle compared to international standards. In addition, the quality of fish for human consumption was assessed.

## Methods

**Figure 1:**
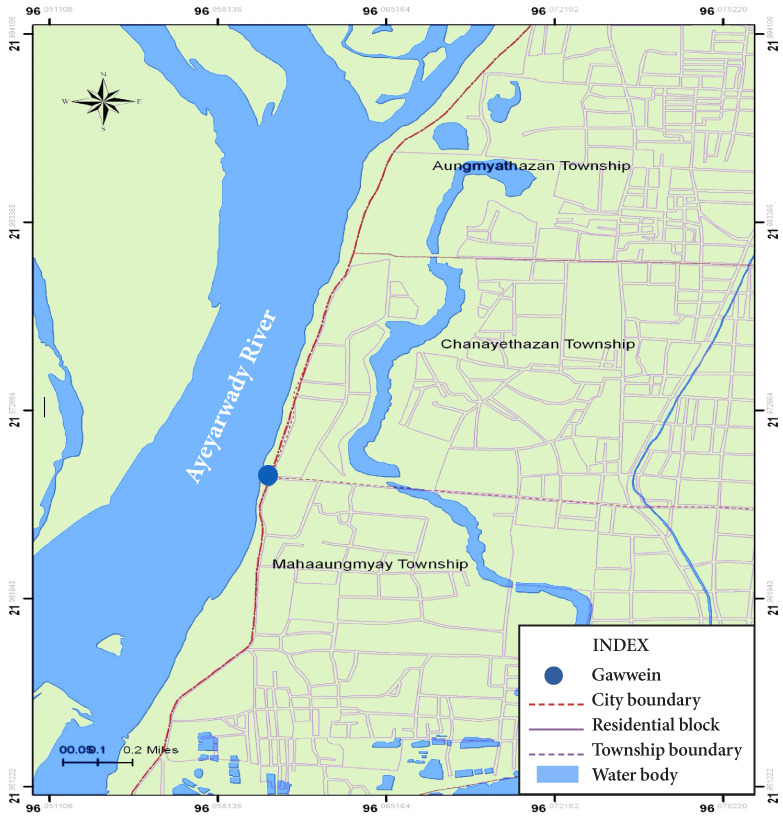
Map of study site (Gawwein fish landing), Mandalay Source: Department of Geography, University of Mandalay^16^

Gawwein fish landing is located in the western part of Mandalay City, on the east bank of the Ayeyarwady River *(Figure 1).* It is the only site that distributes fish caught from the river to markets in Mandalay City. The survey was conducted from July 2010 to September 2011.

### Sample collection

Fish were purchased weekly, directly from local fishermen at Gawwein fish landing site, Ayeyarwady River, Mandalay segment. The collected fish species were identified following the method of Talwar and Jhingran.^17^ Total length and weight were measured and then categorized based on their feeding habits. Based on the stomach contents of fish collected, fish species were classified as herbivores, carnivores and omnivores.

### Sample preparation

The collected fish were washed in tap water to eliminate contamination on the body surface. The fish were beheaded, scaled, gutted and then the muscles were dissected with a cleaned knife. After dissecting, the muscles were sun dried and powdered by blender and pestle and sieved. Finally, dried fish powder was stored in an airtight plastic container. One sample for each species was analyzed for Cd content. For sample preparation, two or three pieces of muscle tissue were used for large specimens and for small fish about 20 fish were pooled.

### Acid digestion method

The acid digestion procedure was used following after Agemian *et al.*^18^ About 5 g of fish homogenate was first digested with 5 ml of concentrated nitric acid (65% analar grade) and 5 ml of concentrated sulphuric acid in borosilicate glass tubes and allowed to react overnight (about 15 hours) at room temperature. The following day, the tubes were placed in a sand bath and heated up to 60°C for about 30 minutes and allowed to cool. After cooling, 10 ml of nitric acid was added and the solution was heated again to about 300°C for 10 minutes. Then, 5 ml of hydrogen peroxide was added and heated until the sample was clear. All samples were totally dissolved. Finally, the samples were cooled again to room temperature and the volume was adjusted to 50 ml of deionized water.

### Metal analysis

The prepared samples were sent to Universities' Research Centre (URC), University of Yangon, to analyze the Cd content. The concentration of Cd was determined by flame atomic absorption spectrometry (Perkin Elmer AAanalyst 800 and Winlab-32 software). The results were expressed as mg/kg or parts per millions (ppm) mg of Cd per kilogram of fish.

### Data analysis

One-way analysis of variance was used to determine statistical differences in Cd content in fish species across different feeding habits (herbivorous, carnivorous and omnivorous fish species) using Statistical Package for the Social Sciences (SPSS) software (p>0.05).

## Results

A total of 25 fish species from Gawwein fish landing in Mandalay were collected and their stomach contents were examined. A total of 1024 fish stomachs were observed, and fish were classified as herbivores, carnivores and omnivores based on their stomach contents *(Table 1).*

**Table 1:** Feeding Habits of Fish Species According to Stomach Contents

**Species**	**Stomach contents**	**Feeding habit**
Notopterus notopterus	Fish, crustaceans and insects	Carnivore
Gudusia variegata	Algae and pieces of aquatic plants and small fish	Omnivore
Labeo calbasu	Algae and aquatic plants	Herbivore
Catla catla	Algae and aquatic plants	Herbivore
Cirrhinus mrigala	Pieces of aquatic plants	Herbivore
Osteobrama belangeri	Algae and aquatic plants	Herbivore
Puntius sophore	Algae and pieces of aquatic plants and small fish	Omnivore
Salmostoma sladoni	Algae and pieces of aquatic plants and small fish	Omnivore
Lepidocephalichthys thermalis	Pieces of aquatic plants and small fish	Omnivore
Botia histrionica	Algae and pieces of aquatic plants and small fish	Omnivore
Sperata aor	Pieces of fish and beetles	Carnivore
Mystus cavasius	Fish, beetles and mud	Carnivore
Mystus leucophasis	Pieces of fish, beetles and mud	Carnivore
Mystus menoda	Fish, beetles and mud	Carnivore
Ompok bimaculatus	Algae and pieces of aquatic plants and small fish	Omnivore
Wallago attu	Fish and insect larvae	Carnivore
Clupisoma prateri	Algae and pieces of small fish	Omnivore
Hemipimelodus jatius	Aquatic insects and fish	Carnivore
Bagarius yarrellii	Pieces of aquatic insects and fish	Carnivore
Xenentodon cancila	Aquatic insects and fish	Carnivore
Parambassis ranga	Aquatic insects and fish	Carnivore
Glossogobius giuris	Aquatic insects and fish	Carnivore
Oreochromis sp.	Algae and pieces of aquatic plants	Herbivore
Channa striata	Aquatic insects and fish	Carnivore
Mastacembelus armatus	Pieces of aquatic plants and insects	Omnivore

According to feeding habits, 5 herbivorous species, 12 carnivorous species and 8 omnivorous species (138 fish species) were analyzed for Cd content. Total length and Cd concentrations for herbivorous, carnivorous and omnivorous fish can be found in Tables 2, 3 and 4 respectively. Cadmium content did not differ significantly across the three fish groups representing different types of feeding habits (p>0.05).

**Table 2:** Cadmium Content of Herbivorous Fish Species from the Gawwein Fish Landing Site

**Scientific Name**	**Local Name**	**Number of specimens**	**Total length (cm)**	**Cd (ppm wt/wt ±SD)**
Catla catla	Nga-gaung-pwa	1	21.7	0.070±0.001
Cirrhinus mrigala	Nga-gyin-phyu	1	38.5	0.071±0.005
Labeo calbasu	Nga-net-pya	1	22.6	0.076±0.010
Osteobrama belangeri	Nga-phant-ma	1	15.1	0.086±0.008
Oreochromis sp.	Tilapia	1	20.3	0.083±0.009

**Table 3:** Cadmium Content of Carnivorous Fish Species from the Gawwein Fish Landing Site

**Scientific Name**	**Local Name**	**Number of specimens**	**Total length (cm)**	**Cd (ppm wt/wt ±SD)**
Notopterus notopterus	Nga-phe	3	13.4–14.7	0.073±0.006
Sperata aor	Nga-gyaung	14	21.8–28.5	0.065±0.015
Mystus cavasius	Nga-zin-yamg-phyu	1	12.7	0.070±0.004
Mystus leucophasis	Nga-nauk-thwa	1	13.0	0.060±0.009
Mystus menoda	Nga-ike	3	17.1–25.8	0.078±0.005
Wallago attu	Nga-butt	1	66.5	0.083±0.007
Bagarius yarrellii	Nga-maung-ma	1	35.5	0.081±0.005
Xenentodon cancila	Nga-phaung-yoe	2	13.2–16.3	0.081±0.013
Hemipimelodus jatius	Nga-yaung	2	8.7–10.5	0.082±0.015
Parambassis ranga	Nga-zin-zat	8	5.4–6.2	0.076±0.012
Glossogobius giuris	Nylon-nga	16	6.8–10.9	0.071±0.023
Channa striata	Nga-yant	1	40.5	0.073±0.007

**Table 4:** Cadmium Content of Omnivorous Fish Species from the Gawwein Fish Landing Site

**Scientific Name**	**Local Name**	**Number of specimens**	**Total length (cm)**	**Cd (ppm wt/wt ±SD)**
Gudusia variegata	Nga-la-bi	22	8.2–10.0	0.084±0.015
Puntius sophore	Nga-khone-ma	10	6.2–83	0.074±0.060
Salmostoma sladoni	Yin-baung-za	24	7.6–10.8	0.082±0.024
Lepidocephalichthys thermalis	Nga-tha-le-doe	14	8.8–16.0	0.074±0.012
Botia histrionica	Nga-shwe-yway	6	11.2–12.5	0.070±0.014
Ompok bimaculatus	Nga-nu-than	1	25.8	0.075±0.018
Clupisoma prateri	Nga-myin-kun-man	1	26.5	0.078±0.008
Mastacembelus armatus	Nga-mway-na-gar	2	19.7–38.6	0.078±0.006

## Discussion

The results of the present study indicate that Cd was detected in all fish samples (total of 25 samples) examined. The lowest Cd level was found in Mystus leucophasis, while the highest was found in Osteobrama belangeri. In Myanmar, Mu reported that the accumulation of Cd in the muscle of Channa striata (Nga yant) in Hinthada township was 0.077 ± 0.025 mg/kg.^19^ Mar reported that the level of Cd in the muscle of Channa striata in the Ayeyarwady River segment, Mandalay Region, was 0.073 ± 0.007 mg/kg.^16^ The present findings agree with the results of these previous studies.

Paudel *et al*. found that the concentration of Cd in Clarias batrachus in fish markets of Kathmandu Valley, Nepal was 0.53±0.33 ppm, while Ahmed *et al*. reported accumulation of Cd in the tissues of Channa striata and Clarias batrachus from the Wang Mengkuang abandoned tin mine, Malaysia of 0.10 ± 0.00 ppm and 0.12 ± 0.01 ppm, respectively.^20^,^12^ The results of the present study were lower than the findings of these two previous studies.

In Myanmar, the source of Cd input to the Ayeyarwady River is not precisely known, but possible sources mentioned in the literature were electroplating and heavy industries. However, no industries of this nature are present in this area. The Bay of Bengal Marine Ecosystem Project, Country report on pollution—Myanmar^13^ states that industries, agriculture and municipalities commonly discharge waste into nearby creeks and rivers, eventually polluting larger water bodies. The growth of industries, increased use of fertilizers and pesticides, urbanization and discharge of municipal waste are increasingly polluting the river system. As a result, some rivers are polluted with residual fertilizers, pesticides and municipal waste. A total of 352 698 tons of chemical fertilizers and 4940 metric tons of pesticides were used in 2009–10. In addition, agricultural chemicals, communities and cities along the river directly dispose untreated raw sewage, municipal wastes, and medical wastes into rivers, polluting the river system.^13^ The Ayeyarwady River is the lifeline of Myanmar and the majority of the country's population is dependent on the river for their survival. The ecology of the river is under serious threat. Fish can accumulate Cd from the water, leading to possible human consumption of Cd-contaminated fish (contaminated food chain).^22^

Information on heavy metal concentrations in fish is important for management of human health risks and pollution control strategies.^17^ Most fish species are at the top of the aquatic food chain and have the potential to accumulate high metal content even in mildly polluted conditions and metals accumulated in the muscle tissue of fish are of great importance due to human health concerns.^18^ An early example of environmental problems due to chronic Cd poisoning (itai-itai disease) occurred in Fugawa, Japan, in 1955.^18^ Moreover, the consumption of a Cd-contaminated rice and fish diet has been shown to cause chronic renal failure in northcentral Sri Lanka.^21^ Manures, fertilizers, and atmospheric precipitation are major sources of Cd leading to Cd accumulation in agricultural soils due to over use of manure and phosphate.^13^,^22^

The maximum permitted level of Cd in fish muscle according to different organizations and guidelines is presented in Table 5. The European Union has set Cd tissue residues of 0.1 ppm fresh weight of marine fish as the criterion for human health protection, whereas the Food and Agriculture Organization/World Health Organization and European Commission limits Cd tissue residues to 0.5 ppm fresh weight of freshwater fish. The Cd content in the muscle of fish studied was above the permissible limit (0.05 mg/kg) set down by the European Commission and the Food and Agriculture Organization/World Health Organization but within the limits of (0.01–0.21 mg/kg) set by the United States Food and Drug Administration and (0.1 mg/Kg) of European Union limit *(Table 5)*.^23^–^26^ It should be noted that no uniform source of guidance or standards for most metal residues in fish tissue is available. There is no single reference for acceptable levels of most metals in marine or freshwater fish, whether self-caught or commercially harvested.

**Table 5:** Maximum Permitted Levels of Cd in Fish Muscle (mg/Kg) According to International Organizations Compared with the Present Study

**Element**	**International guidelines**				**Present Study**
	EU^23^	EC^24^	USFDA^25^	FAO/WHO^26^	
		
Cadmium	0.1	0.05	0.01–0.21	0.05	0.060–0.086

Abbreviations: EC, European Commission; EU, European Union; FAO/WHO, Food and Agriculture Organization/World Health Organization; USFDA, United States Food and Drug Administration.

Cadmium accumulates primarily in the liver and kidneys of vertebrates and not in the muscle tissue and as intestinal absorption is low, biomagnification through the food chain may not be significant.^27^ The present study presents information on Cd content in fish analyzed in the study area and indicates that Cd content did not differ significantly among fish with different feeding habits. The results of the present study suggest that Cd levels in fish are unrelated to the sources of food for fish in the study area.

From the results of this study, it can be assumed that Cd does not presently biomagnify through the food chain. Nevertheless, uptake of Cd by fish has important implications for human exposure to Cd, whether or not significant biomagnification occurs. The concentration of Cd in fish samples across different feeding habits indicate a human health risk for local consumers. The results of the present study suggest that measures should be taken to control the cumulative effects of Cd and to monitor the water quality of the Ayeyarwady River.

## Conclusions

The results of the present study indicate that the high level of Cd in muscle tissues of studied fish species may be due to anthropogenic activities such as municipal waste, and overuse of fertilizers, manures and pesticides on farms along the Ayeyarwady River, as well as domestic wastes. Although the number of samples was limited, the present findings highlight the presence of Cd contamination in fish from the Ayeyarwady River. In terms of public health, fish commonly consumed by the local people in the Mandalay Area may pose significant human health risks due to heavy consumption of Cd-contaminated fish.
